# Quantification of *Leishmania infantum *DNA in females, eggs and larvae of *Rhipicephalus sanguineus*

**DOI:** 10.1186/1756-3305-4-56

**Published:** 2011-04-13

**Authors:** Filipe Dantas-Torres, Maria Stefania Latrofa, Domenico Otranto

**Affiliations:** 1 Dipartimento di Sanità Pubblica e Zootecnia, Università degli Studi di Bari, Valenzano, BA, Italy

## Abstract

**Background**

*Leishmania infantum *is a widespread parasite that affects dogs and humans worldwide. It is transmitted primarily by phlebotomine sand flies, but recently there has been much discussion on the role of the brown dog tick, *Rhipicephalus sanguineus*, as a potential vector for this protozoan. Recent laboratory and field investigations have contributed to this hypothesis, but a proof of the vector capacity of *R. sanguineus *has yet to be provided. Following a recent study suggesting that *L. infantum *passes transovarially from the female tick to her progeny the current study provides new evidence of the transovarial transmission of *L. infantum *in *R. sanguineus*.

**Methods**

Engorged females of *R. sanguineus *were collected from the environment in a dog shelter of southern Italy, where canine leishmaniosis is endemic. In the laboratory, 97 females that successfully laid eggs, their eggs and the originated larvae were subjected to DNA extraction and then tested by a TaqMan-based real time PCR targeting a fragment of the kinetoplast DNA (kDNA) of *L. infantum*.

**Results and conclusions**

*L. infantum *kDNA was detected in engorged females, their eggs and originating larvae, with a parasite load ranging from 1.8 × 10^-4 ^to 10.0 × 10^0^. Certainly, the current study provides further evidence on the passage of *L. infantum *from *R. sanguineus *females to their offspring. The observation of promastigote forms in larvae is necessary to definitively confirm this hypothesis, which would raise interesting questions about the possible role of ticks in the maintenance of *L. infantum *infection among dogs in certain areas.

## Background

*Leishmania *parasites (Kinetoplastida: Trypanosomatidae) are digenetic protozoa responsible for a group of parasitic diseases generally referred to as the leishmaniases. These diseases, most of which are zoonoses, are responsible for a huge burden on public health, causing considerable morbidity and mortality in about 88 countries over the world [1,2]. Among the different clinical forms of the disease, the visceral one is of major importance for being life-threatening and for affecting mainly children and immunodepressed individuals [1,2].

*Leishmania infantum *(synonym, *Leishmania chagasi*) is one of the causative agents of visceral leishmaniasis, an important zoonosis in Europe, Africa, Asia and America [1-4]. This protozoan is primarily maintained in nature by wild reservoir hosts, such as rodents, marsupials, edentates and canids [5]. In the peridomestic transmission cycle, dogs play a role as reservoir hosts for *L. infantum*, mainly because they are quite susceptible to the infection and present a typically heavy skin parasitism [6], which ultimately facilitates the acquisition of the parasites by phlebotomine sand fly vectors (Diptera: Psychodidae), while they are taking a bloodmeal.

Although *L. infantum *is primarily transmitted by phlebotomine sand flies [7], secondary modes of transmission (e.g., transplacental transmission and *via *blood transfusion) have been claimed to exist [8-10]. Recently, it has been demonstrated that day-feeding midges (Diptera: Ceratopogonidae) of the genus *Forcipomyia *can support the development of an undescribed species of *Leishmania *that was originally detected in red kangaroos (*Macropus rufus*) in Australia around eight years ago [11]. Moreover, there has long been speculation about the role of fleas and ticks as vectors of *L. infantum *[12] and recent studies have reinforced this hypothesis [13,14]. Nonetheless, a definitive proof that fleas or ticks can efficiently transmit *L. infantum *from dog to dog under natural conditions has yet to be provided [15].

In a recent study, *L. infantum *kinetoplast DNA (kDNA) was detected in eggs and larvae from infected females, even four months post-inoculation, suggesting the possibility of transovarial passage of the protozoa in *R. sanguineus *[16]. However, the aforementioned study was performed using experimentally infected females, which were artificially inoculated with stationary-phase promastigotes [16]. Undoubtedly, it would be valuable to reassess this hypothesis using naturally infected females. In this perspective, the present investigation was carried out in order to demonstrate the occurrence of transovarial passage of *L. infantum *kDNA in naturally infected *R. sanguineus *ticks. In particular, the research's specific objectives were to detect and quantify the amount of *L. infantum *kDNA present in engorged, wild-collected females, their laid eggs and the originating larvae, using a highly sensitive real time polymerase chain reaction (PCR) protocol.

## Methods

### Collection, identification and rearing of ticks

On 30 April 2009, engorged female ticks (*n *= 100) were collected directly from the environment in a dog shelter located in southern Italy, where *R. sanguineus *is the only tick species present [17]. In the laboratory, the females were rinsed in distilled water and dried with a clean filter paper. The identity of the ticks was immediately determined based on morphology [18].

The engorged females were placed in individual plastic collectors with some holes on the top for allow the air to enter. Then, the vials were placed in an incubator under controlled conditions (26 ± 1°C, 70 ± 10% relative humidity, and scotophase) for the females to lay their eggs. Each female was monitored every day and, after the end of the oviposition period, they and their egg batches (~10 mg) were separated for subsequent DNA extraction, being the remaining eggs left in the incubator. After the eggs were hatched, larvae from 16 females were separated in 10 pools of 10 larvae each. As a rule, females, eggs and larvae were frozen at -20°C until DNA extraction.

### DNA extraction and real time PCR protocol

Genomic DNA was extracted from 97 females (3 females did not lay eggs), eggs and larvae using DNeasy Blood & Tissue Kit (Qiagen, GmbH, Hilden, Germany), in accordance with the manufacturer's instructions. Additionally, and differently from the extraction protocol, all tick specimens were pre-treated by three cycles of freezing (-80°C) and boiling for 10 min, and the extracted DNA was eluted in 50 μl of elution buffer AE (Qiagen).

Real-time PCR for simultaneous detection and quantification of *L. infantum *kinetoplast minicircle DNA was performed using primers LEISH-1 (5'-AACTTTTCTGGTCCTCCGGGTAG-3') and LEISH-2 (5'-ACCCCCAGTTTCCCGCC-3') and TaqMan-MGB probe (FAM-5'-AAAAATGGGTGCAGAAAT-3'-non-fluorescent quencher-MGB) designed by Francino and collaborators [19]. The reaction mixture (20 μl) contained 10 μl of iQ™ Supermix (Bio-Rad Laboratories, Hercules CA, USA), each primer at a concentration of 900 nM, the probe at a concentration of 200 nM, and 2 μl of template DNA. The run consisted of a hot start at 95°C for 3 min and 42 cycles of denaturation (95°C for 10 sec) and annealing-extension (60°C for 30 sec). All assays were carried out in duplicate, with negative (*R. sanguineus *from a laboratory colony) and positive (lymph node tissue from a *L. infantum*-infected dog) controls included in each run. The qPCR was performed in a CFX96™ Real-Time System (Bio-Rad Laboratories, Inc., Hercules CA, USA). The increase in the fluorescent signal was registered during the extension step of the reaction and the data analysed by CFX Manager™ Software Version 1.6 (Bio-Rad).

Parasites were quantified by the comparative Ct method (2^ΔΔCt^) [19]. A 10-fold dilution series of standard DNA from promastigotes (log phase concentration, 1.7 × 10^6 ^parasites/ml) of *L. infantum *(zymodeme MON-1) was used as calibrators, allowing plotting a standard curve, each dilution being tested in triplicate. The limit of detection of the real time PCR was assessed using a serial dilution from 1.7 × 10^-1 ^to 1.7 × 10^-8 ^parasites per reaction. The inter-assay reproducibility was estimated by testing 10-fold serial dilutions, being the experiment repeated 10 times. Results were expressed as the number of parasites per reaction mixture (i.e., 2 μl of template DNA), taking into account the initial concentration and subsequent dilutions used to plot the standard curve.

### Statistical analysis

Positivity rates were calculated as the number of positive samples divided by the number of tested samples multiplied by 100, and expressed as percentages. Additionally, the minimum (number of positive pools/total number of larvae tested) and maximum (number of positive pools/number of larvae in positive pools) positivity rates were calculated for larvae, considering that at least one larva in each positive pool must be positive. Ninety five percent confidence intervals (95% CI) were calculated for each positivity rate. Again, the parasitic load (number of parasites per reaction mixture) in females and eggs were compared using Mann-Whitney U test, differences being considered statistically significant when *P *was 0.05 or less (*P *≤ 0.05). Finally, the parasite loads detected in females and eggs were statistically compared using the Spearman's rank correlation coefficient (*rs*). Statistical analyses were carried out using BioEstat (version 5.0; Mamirauá/CNPq, Belém, PA, Brazil).

## Results

Out of 97 field-collected engorged females of *R. sanguineus *tested by real time PCR for the detection of *L. infantum *kDNA, 25 (25.8%, 95% CI: 17.1%-34.5%) were positive, with a parasite load (mean ± standard deviation: 3.4 × 10^-1 ^± 7.7 × 10^-1^) ranging from 2.9 × 10^-3 ^to 2.8 × 10^0 ^parasites per PCR reaction. In the same way, out of 97 pools of eggs laid by the aforementioned females, 39 (40.2%, 95% CI: 30.5%-50.0%) were positive by real time PCR, with a parasite load (2.8 × 10^-1 ^± 1.6 × 10^0^) ranging from 1.3 × 10^-4 ^to 10.0 × 10^0 ^parasites per PCR reaction.

Out of 160 pools of larvae tested, 93 (58.1%, 95% CI: 50.5%-65.8%) were positive, with a parasite load (2.9 × 10^-3 ^± 1.0 × 10^-2^) ranging from 1.8 × 10^-4 ^to 9.8 × 10^-2 ^parasites per PCR reaction. The overall minimum and maximum positivity rates were 5.9% (95% CI: 4.7%-7%) and 10% (95% CI: 8.1%-11.9%). In particular, among the 93 positive pools of larvae, 56 (60.2%, 95% CI: 50.3%-70.2%) were from eight positive females and 37 (39.8%, 95% CI: 29.8%-49.7%) from eight negative females. Furthermore, 58 positive pools of larvae came from 10 positive pools of eggs whereas 35 positive pools of larvae came from six negative pools of eggs.

No statistically significant difference was found when the parasite loads detected in females and in pools of eggs were compared (Mann-Whitney U test, *P *= 0.706). In the same way, no significant correlation was found between the parasite load detected in females and pools of their eggs (*rs *= -0.03, *P *= 0.718).

**Figure 1:**
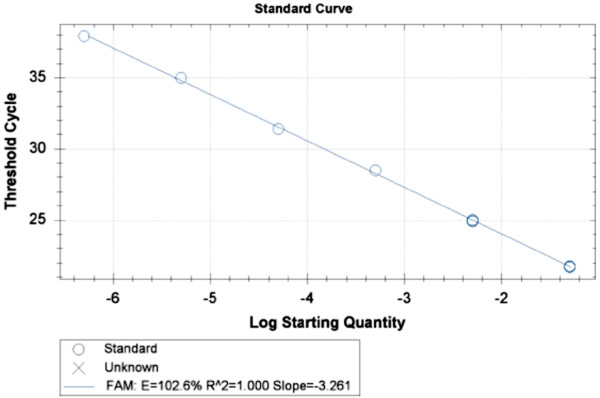
**A standard curve of the log concentration of *L. infantum *DNA**. Standard curve obtained from serial dilutions of *L. infantum *DNA, expressed herein as the number of parasites per reaction mixture (i.e., 2 μl of template DNA). Each point was tested in triplicate. Slope = -3.261; Efficacy = 102.6%; R^2 ^= 1.000.

The real time-PCR protocol used in this study was able to detect very low amounts of *L. infantum *kDNA, with a limit of detection of 1.7 × 10^-6 ^parasites per PCR reaction (data not shown). Figure 1 presents the standard curve, slope and efficacy of a typical experiment. The mean Ct (cycle threshold) values for positive females, eggs and larvae were 35, 36 and 36, respectively.

## Discussion

The present study demonstrates that *L. infantum *kDNA is passed from the engorged female tick to her originating larvae. Because no statistically significant differences were found in relation to the estimated numbers of parasites detected by real time PCR in females and their eggs, it appears that the amount of *L. infantum *kDNA and, most likely, the number of parasites present in females, remained relatively stable in her progeny. This finding was remarkable, especially if one considers that only a small amount of the eggs from each female was tested by real time PCR. This suggests that the amount of *L. infantum *kDNA would probably be greater if more eggs had been tested.

Concerning the correlation between the positivity of engorged females, eggs and larvae, different situations were documented: (I) female positive → eggs positive → larvae positive; (II) female positive → eggs negative → larvae positive; (III) female negative → eggs positive → larvae positive; (IV) female negative → eggs negative → larvae positive; and (V) female negative → eggs negative → larvae negative (data not shown). The absence of correlation between the positivity in engorged females, eggs and larvae might be explained by the presence of PCR inhibitors in engorged females (e.g., situations III and IV) and by the fact that not all eggs or larvae were tested by real time PCR. Thus, in the cases II and IV, it is probable that eggs and/or larvae had been positive if additional samples of eggs and larvae had been tested. Although minimal, the possibility of contamination in one of these egg and larval pools cannot be completely ruled out.

An early study conducted in France failed to demonstrate the occurrence of transovarial passage of *L. infantum *in *R. sanguineus *[20]. More recently, an experimental study using a high sensitive real time-PCR protocol reported the detection of kDNA in artificially infected engorged females, their eggs and the originating larvae [16]. These findings are corroborated by the present study, which included field-collected females that had fed on dogs naturally infected by *L. infantum *instead of artificially infected engorged females used in the previous study [16]. As such, these results reinforce the hypothesis of the occurrence of transovarial passage of *L. infantum *in *R. sanguineus*. The confirmation of this theory would provide a strong supporting evidence for the participation of *R. sanguineus *in the maintenance of *L. infantum *in nature.

Further research to assess the presence and viability of *L. infantum *in unfed larvae is needed in order to fully assess the occurrence of transovarial transmission of *L. infantum *in *R. sanguineus*. Indeed, this hypothesis needs to be confirmed by the visualization of promastigotes in unfed larvae or by the detection of *L. infantum *RNA expression, in order to substantiate the present of live parasites in ticks. Accordingly, *L. infantum *RNA has recently been detected in ticks collected from naturally infected dogs [21], confirming definitively the viability of the parasites in the ticks and supporting the participation of *R. sanguineus *as an invertebrate host of *L. infantum*. In fact, *R. sanguineus *is a competent definitive host for many pathogens, including the protozoa *Babesia vogeli *and *Hepatozoon canis *[22].

In conclusion, the present investigation reports the detection and quantification of *L. infantum *DNA in field-collected engorged females, eggs and larvae, providing further evidence on the occurrence of transovarial passage of *L. infantum *in *R. sanguineus*, although this hypothesis has yet to be proven. This finding suggests that the hypothesis of ticks as vectors of this protozoan between dogs is reasonable and needs to be finally confirmed by finding of promastigote forms in these arthropods.

## Competing interests

The authors declare that they have no competing interests.

## Authors' contributions

FDT collected, identified and reared the ticks in the laboratory, contributed with data analysis and interpretation and wrote the first draft of the manuscript. MSL run the real time PCRs and contributed with data analysis and interpretation. DO contributed with data analysis and interpretation and revision of the manuscript. All authors read and approved the final version of the manuscript.
